# Highly flexible and ultrathin electromagnetic-interference-shielding film with a sandwich structure based on PTFE@Cu and Ni@PVDF nanocomposite materials

**DOI:** 10.1039/d2ra05439f

**Published:** 2022-10-17

**Authors:** Bingzhi Guo, Jianying Liang, Jiongfeng Chen, Yun Zhao

**Affiliations:** Beijing Institute of Technology Zhuhai 519088 P. R. China zhaoyun@bit.edu.cn; Guangxi University Nanning 530004 P. R. China; School of Chemistry and Chemical Engineering, Beijing Institute of Technology Beijing 100081 P. R. China

## Abstract

Light and flexible electromagnetic-interference-shielding materials are of great significance to control electromagnetic pollution and protect the human body and other nearby equipment or systems. In this study, a film of polytetrafluoroethylene wrapped with copper (PTFE@Cu) was prepared by depositing Cu using electroless plating on the surface of a microporous PTFE film modified by dopamine. A Ni@PVDF membrane was fabricated by casting a suspension of Ni nanochains in PVDF. The two kinds of films were hot-pressed into an ultrathin and efficient electromagnetic-shielding film with a sandwich structure. PTFE and PVDF provided high flexibility to the composite film, while the metal-wrapped polymer fiber structure gave the film an excellent electromagnetic-shielding efficiency, and the Ni nanochains and laminated hot-pressing process further enhanced the shielding ability of the film. Through these combined effects, the conductivity of the composite film reached 1117.57 S cm^−1^ while the thickness was only about 80 μm, and the average shielding efficiency in the X-band range was as high as 57.16 dB with absorption accounting for about 67.2% of the total shielding. At the same time, the composite film had high strength and flexibility, and the tensile strength could reach 43.49 MPa. Even after bending 1000 times, the conductivity could still be maintained at 174.55 S cm^−1^, while the average shielding effectiveness in the X-band range was retained at 44.29 dB. The film has great latent applications in flexible devices and portable wearable intelligent devices.

## Abstract 2

Light and flexible electromagnetic-interference-shielding materials are of great significance to control electromagnetic pollution and protect the human body and other nearby equipment or systems.

## Graphical abstract

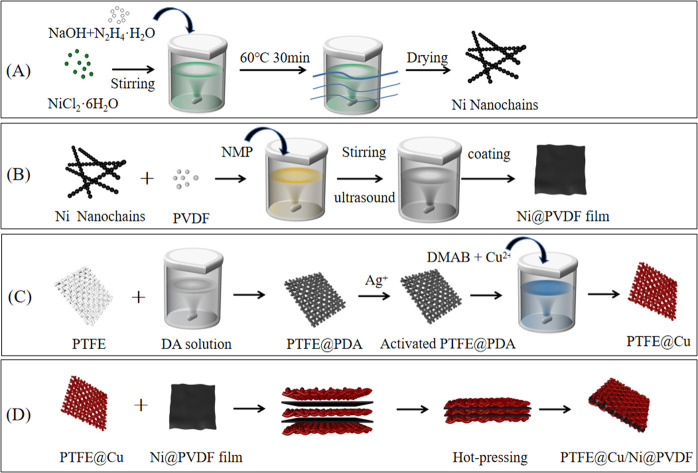

## 1. Introduction

With the widespread use of electronics, electronic circuits and electrical components are gradually progressing towards miniaturization, digitization, light weight, and integration. However, the large and extensive utilization of such electronic equipment not only facilitates the lives and work of human beings but also produces a lot of electromagnetic interference wave pollution, which can have a serious impact on the performance of mobile phones, computers, hospital equipment, and other equipment, and can even cause serious harm to humans and other organisms.^1–5^ Therefore, there is an essential and urgent need to develop a protection system with an electromagnetic interference (EMI) shielding function for next-generation smart electronics. The main disadvantages of the shielding materials currently used are their large thickness and poor mechanical properties, which limit their application.^6–8^ Light and flexible EMI-shielding materials are of great significance to reduce electromagnetic pollution and protect life and other nearby equipment or systems.^9,10^

EMI-shielding materials reflect or absorb incident electromagnetic waves through electrical loss, dielectric loss, and magnetic loss. Metal materials have a good EMI-shielding effect (SE) in high and low electromagnetic fields and electrostatic fields due to their good conductivity and permeability; however, all of them have the disadvantages of high density, easy corrosion, and difficult processing.^11–13^ In contrast, polymer matrix composites with excellent conductivity can overcome the shortcomings of metal materials, have number of advantages, such as corrosion resistance, low density, splendid chemical stability, thermal steadiness, and mechanical constancy, which can better meet the needs of aerospace, electronic communication, and other fields, and are considered to be a kind of EMI-shielding materials with great development potential.^14–16^ Polymer matrix electromagnetic-shielding films are usually obtained by compounding fillers with conductive functions (such as graphene,^3^ MXene,^6,17^ CNT,^10^ and SiO_2_^18^) with polymers (PVDF,^19^ PVA,^4^ PDMS,^20^ PBT,^18^ PPy,^21^*etc.*) and then form films by casting, impregnation, extrusion, calendaring, or other film-forming methods and adjusting the SE by controlling the thickness of the film. In addition, loading nanomaterials with an electromagnetic-shielding function on natural polymer materials (such as cotton^22^ and wool^23^) is also of great significance for the preparation of flexible wearable devices. However, due to the grievous impedance mismatch between solids and air, incoming electromagnetic waves are mainly reflected rather than absorbed, resulting in secondary EMI pollution in the surroundings.^24^ The preparation of light polymer conductive composite EMI-shielding materials with a porous structure has turned into a hot spot in recent years. A porous structure can not only just further cut down the material density but also enhance the multiple scattering/reflection reduction of electromagnetic waves in the pores so that electromagnetic waves entering the material fall into the “labyrinth structure” and dissipate in the form of heat so as to improve the absorption performance of materials to electromagnetic waves.^1,8,16,25–27^ In addition, based on the electromagnetic wave loss theory, the combination of a dielectric dipole and magnetic dipole and the addition of magnetic nanomaterials, such as Ni, Co, and Fe_3_O_4_, in to polymer matrix composites is considered to be a promising solution.^2,10,28^ Zeng *et al.* devised a nylon nanofiber microporous film wrapped with metal Cu, and reported that the EMI SE reached 53 dB in a wide frequency range when the film thickness was only 2.5 μm, but the tensile strength was less than 20 MPa.^1^ Zhendong Dai *et al.* prepared a lightweight multilayer EMI-shielding material consisting of a porous foam with Cu–Ni alloy and CNTs, and its EMI SE achieved 47.5 dB in the X-band range, but the material was not flexible.^29^ CuNW@G core–shell aerogels exhibited an average validity of 52.5 dB in the X-band, but their mechanical properties were not satisfactory.^30^ CuNW/PS nanocomposite powders were formulated by solution processing and displayed an EMI SE of 35 dB in the X-band, however the contribution of absorption to the overall shielding was only about 54%.^31^

As is well known, the structure of composite materials and their conductivity have a very intense bond, which naturally has a conspicuous impact on their EMI SE.^32^ Luyang Liang *et al.* reported electromagnetic-shielding films based on PVDF containing oriented graphene nanosheets and Ni nanowires with heterogeneous alternating multilayer structures, which showed a strong EMI SE of 43.3 dB when the thickness was about 0.5 mm^2^. Xiuxiu Jin *et al.* assembled a heterogeneous PVA/MXene film with an alternating multilayered structure, and the film expressed a maximum EMI SE of 44.4 dB when the thickness was 27 μm.^33^ Yongqiang Guo *et al.* fabricated an oriented GNPs/PS composite, which exhibited an EMI SE of 33 dB.^34^ Combining metal-wrapped polymer fibers with magnetic metals to prepare composite films with a sandwich structure would offer obvious advantages in improving the EMI shielding. With the exception of the EMI performance, flexible devices and portable wearable intelligent devices require certain integrated properties, including high conductivity, a light weight, and a high resistance to mechanical deformation.^1^ Ultrathin electromagnetic-shielding films with flexibility are thus in great demand.

In this work, a PTFE microporous membrane was modified by dopamine, and then a layer of Cu was plated on its surface by electroless plating, and a Cu plating film with excellent EMI-shielding performance was subsequently obtained (PTFE@Cu). PVDF powder and Ni nanochains were mixed evenly in NMP solution by magnetic stirring, and then the mixture was coated on a glass surface and heated to remove the solvent to obtain the Ni@PVDF film. The ultrathin and flexible EMI-shielding film with a sandwich structure (PTFE@Cu/Ni@PVDF) was prepared from PTFE@Cu and Ni@PVDF films by a further laminated hot-pressing process. It showed overall shielding of about 67.2% at a thickness of 80 μm. Simultaneously, the tensile strength of the film reached 43.49 MPa. Therefore, it shows gigantic latent promise for use in flexible devices and portable wearable intelligent devices.

## 2. Experimental section

### 2.1. Materials

PTFE hydrophilic microporous film (PTFE film), was purchased from Changzhou Jinchun Environmental Protection Technology Co., Ltd. Dopamine hydrochloride, 98%, was obtained from Hefei Bomei Biotechnology Co., Ltd. Tris HCl, molecular weight 157.64, 99.5%, was supplied by Beijing Yami Biotechnology Co., Ltd. Sodium hydroxide (NaOH) and ethanol were purchased from Tianjin Damao Chemical Reagent. Copper chloride (CuCl_2_), nickel chloride hexahydrate (NiCl_2_·6H_2_O), silver nitrate (AgNO_3_), dimethylamino borane (DMAB), and ethylenediaminetetraacetic acid disodium salt (EDTA) were obtained from Guangdong Wengjiang Chemical Reagent Co., Ltd. Hydrazine monohydrate (N_2_H_4_·H_2_O, 80 wt%), boric acid (H_3_BO_3_), and *N*-methylpyrrolidone (NMP) were supplied by Sinopharm Chemical Reagent Co., Ltd. PVDF powder, Arkema HSV900, was purchased from Dongguan Shunjie Plastic Technology Co., Ltd. All the chemicals were used directly as received.

### 2.2. Preparation of the test samples

**Fig. 1:**
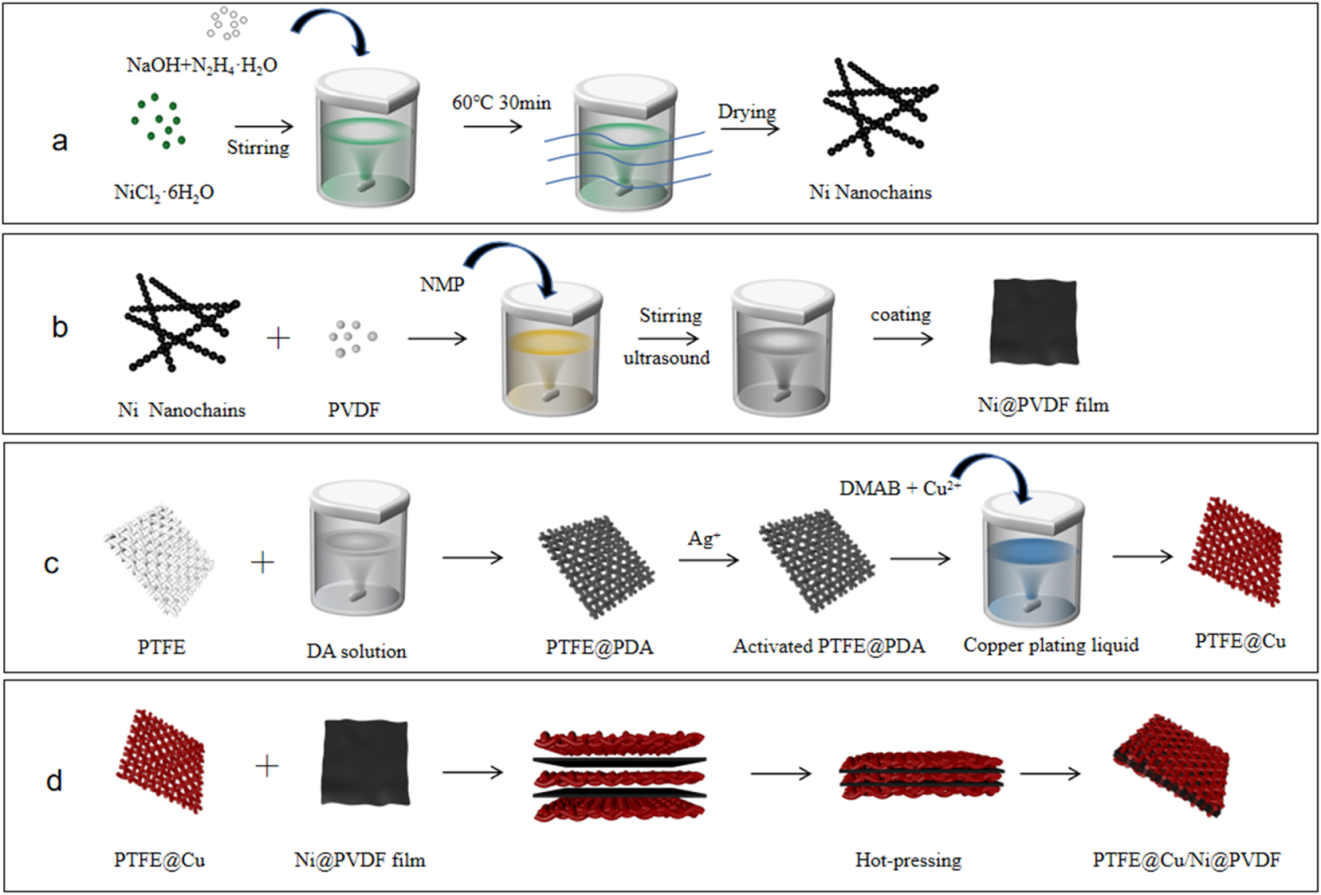
Schematic illustration for the preparation of (a) Ni nanochains, (b) Ni@PVDF film, and (c) PTFE@PDA, PTFE@Cu, and (d) PTFE@Cu/Ni@PVDF films.

Ni nanochains were prepared by a liquid-phase chemical reduction, as shown in Fig. 1a.^35^ First, 30 ml deionized water and 30 ml ethanol were mixed into a solution, and then 0.3 g NiCl_2_·6H_2_O was dissolved in it, with 0.5 g NaOH solid particles then added and the solution agitated until the particles had completely dissolved. Next, 6.0 ml N_2_H_4_·H_2_O solution was added into the system, and the solution reacted in a water bath for 30 min at 60 °C without stirring, with the bath then placed in a 0.3 T parallel magnetic field. Black flocs were obtained floating on the surface of the mixed solution, that is, Ni nanochains, which were washed with deionized water and ethanol 3–5 times. Lastly, the system was dried in a vacuum at 60 °C for 1 h.

Next, 1.0 g of PVDF powder and 0.2 g Ni nanochains were added to 9.0 ml NMP with magnetic stirring for 5 h, and then ultrasonic treated for 10 min to obtain a uniformly dispersed PVDF/Ni nanochains mixture. A coating rod was used to evenly coat the mixed liquid on to a glass plate, which was then put into a vacuum at 60 °C for 5 h to completely dislodge the dissolvent, and then the Ni@PVDF film was obtained (Fig. 1b).

The preparation processes of the PTFE@PDA, PTFE@Cu, and PTFE@Cu/Ni@PVDF films are shown in Fig. 1c and d. The PTFE film was soaked in ethanol for 30 min to remove the oil on its surface, and then it was ultrasonicated for 3 min in deionized water, and then dried at 80 °C for 15 min. Tris HCl was configured as a 10 mM L^−1^ solution, and then dopamine hydrochloride powder was dissolved in the system and the pH was adjusted to about 8.5 with NaOH to obtain 2 g L^−1^ dopamine solution. The PTFE film was immersed in dopamine solution and agitated at room temperature continuously for 24 h. After that, the film was taken out and ultrasonically cleaned in deionized water for 3 min to obtain the dopamine-modified PTFE film (PTFE@PDA), which was then soaked in 0.5 M L^−1^ AgNO_3_ solution for 1 h to obtain activated PTFE@PDA.

For the Cu plating film, 1.71 g CuCl_2_, 1.71 g H_3_BO_3_, and 4.86 g EDTA were dissolved in 250 ml water and mixed into an uniform solution, and then the pH was adjusted to 6.5–7 with NaOH solution. The activated PTFE@PDA was immersed vertically in the system, and ultrasonicated for 5 min to fully wet the sample surface and remove the bubbles on the film surface and inside. Then, the temperature of the system was raised to about 70–80 °C, and DMAB (15 ml, 10% aqueous solution) was added to the liquor with mixing while dropping in the solution. The reaction lasted for 1 h and the Cu plating film (PTFE@Cu) was obtained. The film was ultrasonically cleaned with deionized water for 3 min and air dried at room temperature.

Three pieces of PTFE@Cu films and two pieces of Ni@PVDF films were stacked alternately and sandwiched between two stainless steel plates, which were then hot-pressed for 10 min with a hot press under 25 MPa pressure and 180 °C to obtain the final sample of composite film (PTFE@Cu/Ni@PVDF), as shown in Fig. 1d.

### 2.3. Characterization and measurements

FTIR spectroscopy was performed on a Bruker VERTEX-70 spectrometer. The XRD patterns were characterized using a Shimadzu XRD-6000 X-ray powder diffractometer (Cu Kα radiation, *k* = 0.15406 nm). An ST2253 digital four probe tester was used to test the resistivity of the sample at room temperature; the hysteresis loops of the samples were obtained by using a vibrating sample magnetometer (VSM, LakeShore7404). The tensile properties of the samples were tested by an electronic universal testing machine (Labthink C610H), and the average value of the five samples was taken. A bending test was next performed, whereby the composite film was bent 180° forward and backward along the center of the measured point and maintained for 10 s, which was recorded as a single bending.^36^ The surface topography and structure of the different materials were obtained by using a Zeiss MERLIN high-resolution field-emission scanning electron microscopy (SEM) system. An EDS (Oxford) system was applied to analyze the elemental distribution of Cu and Ni. The EMI SE of the films was measured using a network vector analyzer (Agilent E5071C) based on GJB 6190-2008. The reflection coefficient (*R*), transmission coefficient (*T*), absorption coefficient (*A*), total SE (SE_T_), reflection SE (SE_R_), and absorption SE (SE_A_) were calculated by the *S*-parameter (*S*_11_ and *S*_21_), which was obtained by a network vector analyzer directly. The calculation process was carried out through the following formula; also as a general rule, when SE_T_ > 15 dB, multiple reflection efficiency can be neglected.^2,7,8^

1 *R* = |*S*_11_|^2^*T* = |*S*_21_|^2^

2 *A* = 1 − *R* − *T*

3 SE_R_(dB) = −10 lg(1 − *R*)

4 SE_A_(dB) = −10 lg(*T*/(1 − *R*))

5 SE_T_(dB) = SE_R_ + SE_A_ + SE_M_

## 3. Results and discussion

### 3.1. Characterization of Ni nanochains

**Fig. 2:**
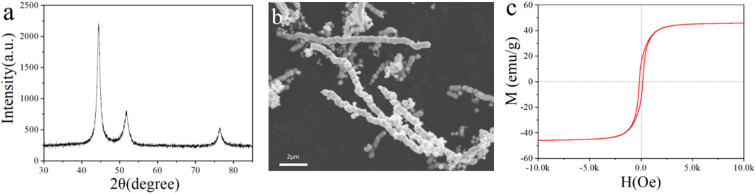
(a) XRD pattern, (b) SEM image, and (c) VSM curve of the Ni nanochains.

The crystal structure and morphology of Ni nanochains were obtained by XRD and SEM techniques. From Fig. 2a, we can see clearly the peaks corresponding to the (111), (200), and (220) crystal planes of the Ni face-centered cubic system, which were 2*θ* = 44.5°, 51.8°, and 76.4°, respectively.^2,37^ The peaks of the spectrum were sharp and no impurity peak was found, indicating the as-prepared Ni nanochains had high purity. It can be seen from the SEM of the Ni nanochains (Fig. 2b) that their diameters and length were approximately 500 nm and about several tens of microns, respectively. Such a one-dimensional chain structure can help its tip receive more electromagnetic waves, which is conducive to decreasing the electromagnetic waves.^38^Fig. 2c shows the hysteresis loop of the Ni nanochains, which presents the criterion ferromagnetic *S*-curve with a coercivity of 170 Oe and a saturation magnetization (*M*_s_) of 45.4 emu g^−1^; these results demonstrate that the as-prepared Ni nanochains had a strong magnetic loss ability.

### 3.2. Morphology and structure of the films

**Fig. 3:**
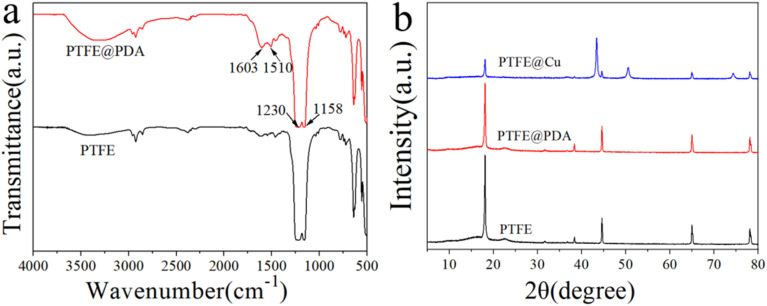
(a) FTIR spectra of PTFE and PTFE@PDA and (b) XRD patterns of PTFE, PTFE@ PDA and PTFE@Cu.

In order to confirm the interaction between dopamine and PTFE, FTIR spectroscopy analysis was performed and the results are illustrated in Fig. 3a. The absorption peak of –OH stretching vibration appeared at about 3300 cm^−1^, which was a result of hydrophilic modification. The strong peaks at 1158 and 1230 cm^−1^ corresponded to stretching vibrations of the C–F bond, which are typical absorption peaks of PTFE.^39^ In the spectrum of PTFE@PDA, the peak at 1510 cm^−1^ was related to the superposition of the C=C skeleton vibration of the benzene ring in PDA, while the 1603 cm^−1^ peak was attributed to the bending vibration of N–H of PDA. These peaks demonstrate that dopamine had polymerized into polydopamine on the surface of the PTFE film.^40,41^Fig. 3b gives the XRD patterns of different films. The PTFE film showed a sharp peak at 2*θ* = 18.1°, which is the characteristic diffraction of PTFE.^37^ When the surface of the PTFE film was modified by dopamine, the characteristic diffraction peak at 2*θ* = 18.1° was weakened, because the polymerization of dopamine on the surface reduced its ability to scatter X-rays, which is consistent with the conclusion from the FTIR study. After plating Cu on the surface of PTFE@PDA, the diffraction peaks in the PTFE@Cu XRD spectrum at 2*θ* = 43.4°, 50.5°, and 74.3°, corresponding to the (111), (200), and (220) crystal planes of copper,^37,42^ respectively, indicated that Cu had been precipitated on the surface of PTFE@PDA. Meanwhile, the characteristic diffraction peak of PTFE at 2*θ* = 18.1° in PTFE@Cu became weaker obviously, although it could still be observed, because the adhesion of PDA and Cu on the surface of PTFE reduced the scattering ability of PTFE to X-rays. In addition, it was found that there was no diffraction peak of CuO or Cu_2_O in the spectrum through comparison, indicating that during Cu plating, the reduced deposition on the surface of the film was pure Cu.

**Fig. 4:**
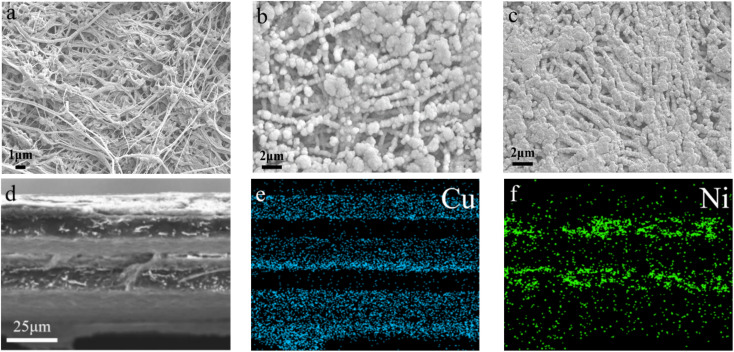
SEM images of the PTFE film (a), PTFE@Cu (b), and PTFE@Cu/Ni@PVDF (c). (d) Cross-section SEM image of PTFE@Cu/Ni@PVDF and elemental mapping of Cu (e) and Ni (f).

Fig. 4 presents the morphology of the films characterized by SEM. The picture in Fig. 4a clearly indicates that the surface of the PTFE film comprised a 3D network structure composed of fibers formed by stretching PTFE particles, whereby porous structures were formed between the fibers with complex structures, such as a network connection, pore bending, and hole inlay. After electroless Cu plating, the surface of PTFE@PDA was completely covered by Cu metal (Fig. 4b). PDA has certain reducibility and can reduce silver ions in solution into silver nanoparticles, but not enough to reduce Cu ions.^43^ Therefore, there were silver nanoparticles on the surface of the activated PTFE@PDA. In the process of copper deposition, these nanoparticles were used as the catalytic centers, where the surrounding copper ions were reduced to elemental copper by DMAB, whereby Cu took these as crystallization centers and gradually grew into large particles. Finally, a dense metal Cu layer with a certain thickness was deposited on the surface of PTFE@PDA.^44^ After hot compression, the Cu layer on the interface of PTFE@Cu/Ni@PVDF was pressed more densely, and some areas were even connected so that the fiber structure could not be seen (Fig. 4c), which is of great benefit for improving its conductivity and EMI SE. Fig. 4d presents a cross-sectional view of PTFE@Cu/Ni@PVDF after a liquid-nitrogen-embrittlement fracture, where it can be distinctly seen that there were three layers of PTFE@Cu sandwiches with two layers of Ni@PVDF matrix, and the thickness was about 80 microns. Fig. 4e and f present the distribution maps of Cu and Ni elements in the cross-section in Fig. 4d, where blue represents the distribution of Cu elements, so that the Cu deposited on the PTFE film can be seen clearly, while the green dots are the distribution of Ni nanochains in the PVDF layer, through which it can be seen that the distribution was slightly agglomerated.

### 3.3. Electromagnetic-interference-shielding property

**Fig. 5:**
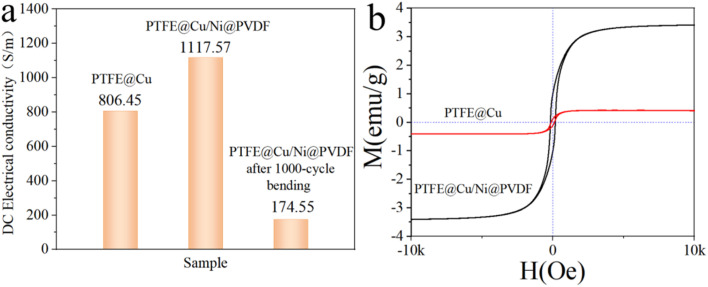
(a) DC electrical conductivity of different films and (b) magnetic hysteresis loops of PTFE@Cu and PTFE@Cu/Ni@PVDF.

Electrical conductivity is a basic characteristic of EMI-shielding materials, which reflects the internal ability of materials to shield electromagnetic waves. Fig. 5a reveals the DC electrical conductivity of different films at room temperature. The conductivity of the obtained PTFE@Cu was 806.45 S cm^−1^. After being prepared as PTFE@Cu/Ni@PVDF, the conductivity further reached 1117.57 S cm^−1^, indicating that the PTFE@Cu/Ni@PVDF film had excellent conductivity. Basically, the enhancement of conductivity could attributed to the denser Cu layer on the surface after hot-pressing.

Through monitoring the relationship between the magnetization variation (*M*) and applied magnetic field (*H*) (Fig. 5b), we could obtain information about the magnetism of the films of PTFE@Cu and PTFE@Cu/Ni@PVDF. The hysteresis loop of the PTFE@Cu sample was almost a straight line, and there was nearly no induced magnetic field when the applied magnetic field increased, indicating that the material was almost non-magnetic. PTFE@Cu/Ni@PVDF showed an S-shaped hysteresis loop similar to that of ferromagnetic materials, and its *M*_s_ was 3.41 emu g^−1^, which was significantly increased compared with that of PTFE@Cu (0.415 emu g^−1^). According to the formula *M*_s_ = *φ* × ms (where *φ* represents the volume fraction of particles and ms means the saturated magnetic moment of a single particle),^45^ the *M*_s_ of PTFE@Cu/Ni@PVDF was chiefly decided by the volume fraction of Ni nanochains. Obviously, the *M*_s_ of PTFE@Cu/Ni@PVDF was fantastically elevated owing to the introduction of Ni nanochains, which is of vital importance for the magnetic loss of EMI shielding.

**Fig. 6:**
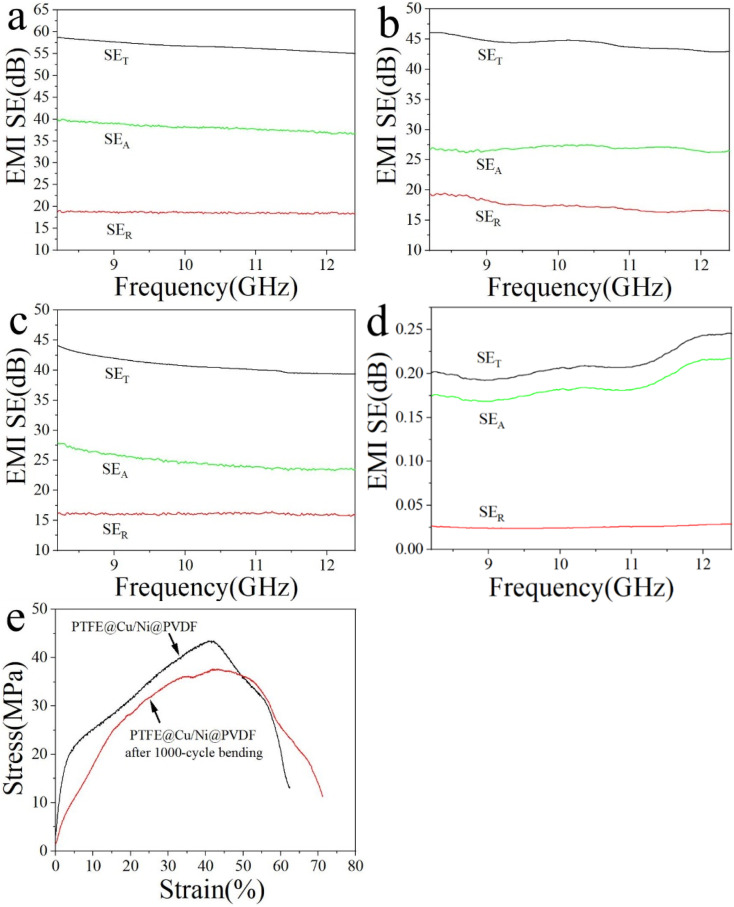
EMI SE of PTFE@Cu/Ni@PVDF before (a) and after 1000 cycle bending (b), PTFE@Cu (c) and Ni@PVDF (d). Tensile curve of PTFE@Cu/Ni@PVDF before and after bending for 1000 cycles (e).

Fig. 6 discloses the EMI-shielding performance of PTFE@Cu and PTFE@Cu/Ni@PVDF films in the X-band (8.2–12.4 GHz). EMI-shielding mechanisms generally consist of reflection (SE_R_), absorption (SE_A_), and multiple internal reflection loss.^1,8^ An effective microstructure scheme is extremely important for high EMI shielding. The PTFE@Cu/Ni@PVDF we designed was arranged in alternating layers of PTFE@Cu and Ni@PVDF. Such a sandwich-structured microporous film takes advantage of the polymer nanofibers and nanometal and their interface interactions, coupled with an inherent porous structure, whereby the synergistic effect enhances the local absorption and multiple reflection of the membrane material. Cu-coated polymer nanofibers can provide a large number of carriers, which can transfer freely within the copper layer, and accelerate the polarization of the interface formed by the conductive copper shell and the insulating polymer core.^1,8^ The polarized interfaces can effectively absorb incident electromagnetic waves because of their high charge-storage capacity.^1^ At the same time, thanks to the multiple reflections of electromagnetic waves at each interface, the dissipative properties of PTFE@Cu/Ni@PVDF were further enhanced.^8^ The number of multiple reflections and scattering were greatly increased due to the huge amount of porous structure in PTFE@Cu/Ni@PVDF, which could effectively improve the shielding performance. Ni nanochains have high conductivity, in addition to lattice vibration, and they also vibrate in the form of particles as a whole. Therefore, Ni nanochains have a smaller electron free path than conductors, and their magnetic domain is smaller than some strong magnetic substances, sometimes even in a single-domain structure, so that they can produce a large hysteresis loss to electromagnetic waves and have a good shielding effect to high- and low-frequency magnetic fields.^46^ Metal nanochains and polymer fibers can also induce multiple internal reflections due to their different electrical properties.^47^ All these factors led to the incoming electromagnetic wave to be dissipated in the limited 3D space of PTFE@Cu/Ni@PVDF due to continuous reflection and re-reflection, therefore, the EMI SE mainly came from the absorption of electromagnetic waves. It can be seen that the as-obtained PTFE@Cu/Ni@PVDF not only showed a great EMI SE, where the average SE_T_ in the X-band reached 56.71 dB (Fig. 6a), but also possessed a high SE_A_ value, where the average value of SE_A_ was 38.11 dB, implying that 67.2% of the electromagnetic waves were assimilated. Especially, after 1000 cycles of bending, the conductivity could still be maintained at 174.55 S cm^−1^ (Fig. 5a), while its average EMI SE could still be maintained at 44.29 dB in the X-band (Fig. 6b); these data are far beyond the acceptable value of the industry by 20 dB, equivalent to 99% of the damping of incoming electromagnetic waves.^1^ By comparison, the average SE_T_ value of PTFE@Cu in the X-band was 40.81 dB (Fig. 6c). At the same time, the average SE_T_ value of Ni@PVDF in the X-band was 0.22 dB, while the average value of SE_A_ was 0.19 dB as shown in Fig. 6d; therefore 86.4% of the EMI SE of the film came from the absorption of Ni nanochains, considering that PVDF has almost no such ability. However, the SE of the film was far different from that of PTFE@Cu/Ni@PVDF, which shows that the sandwich composite film is helpful for obtaining satisfactory results. Generally speaking, the alternating sandwich porous-structured layers resulting in multiple reflections, excellent electrical conductivity, and magnetic loss caused by Ni nanochains enable the as-prepared PTFE@Cu/Ni@PVDF to have an outstanding EMI SE. Fig. 6e shows the tensile curve of the PTFE@Cu/Ni@PVDF film before and after bending for 1000 cycles, where the tensile strength of the composite film could reach 43.49 MPa before bending, and the elongation at break reached 62.4%, while after 1000 cycles of bending, the tensile strength could still be retained at 37.57 MPa, while the elongation at break exceeded 71.2%. The as-prepared composite film had good tensile properties, toughness, and ductility. Some reported lightweight electromagnetic-shielding materials are shown in Table 1, including various kinds of materials, such as CNT, graphene, Mxene, and their polymer composites, which clearly show that, in our work, the composite film with a sandwich porous structure constructed by laminated hot-pressing had a high SE and conductivity, and good prospects for application in flexible devices and portable wearable intelligent devices.

**Table 1:** Some reported works on light and thin EMI-shielding composites

Samples	Thickness (mm)	EMI SE (dB)	Conductivity (S cm^−1^)	Ref.
Cu NMs	0.0025	53	5983	1
Ni@G-P films	0.5	43.5	0.768	2
Ti_3_C_2_T_*x*_/PEDOT:PSS	0.007	55.4	2900	6
H–AgNW/cellulose	0.2	40	33.69	7
Gn-SiCNW/PVDF	1.2	32.5	2.3 × 10^−6^	9
PEDOT:PSS/WPU films	0.15	62	77	15
PVDF/CNT/graphene films	0.25	36.45	10^−3^	19
CNT–MLGEP	1.6	47.5	1.18	26
MCMBs/MWCNTs	0.5	80	8.03	28
Cu–Ni–CNT composite	1.5	47.5	—	29
(CuNW)/polystyrene (PS)	0.21	35	—	31
CNTs/cellulose film	0.15	35	20	32
PVA/MXene	0.027	44.4	716	33
BBF@PANI	0.4	45	8.99	47
PTFE@Cu/Ni@PVDF film	0.08	57.16	1117.57	This work

## 4. Conclusions

In this paper, a high-efficiency electromagnetic-shielding film with a sandwich porous structure was successfully formulated by a laminated hot-pressing process. The surface of the polymer fiber could be effectively Cu plated after being modified by polydopamine, whereby the metal-wrapped polymer fiber structure gave the film excellent EMI SE. Ni nanochains enhanced the magnetic loss of the material. Combined with electromagnetic loss, the film was endowed with an excellent EMI SE. The conductivity of the sandwich-structured film reached 1117.57 S cm^−1^, and the average shielding efficiency reached 56.71 dB in the X-band, where the contribution of absorption to the overall shielding was about 67.2%. PTFE and PVDF provide high flexibility and toughness to the composite film, such that when it was bent 1000 times, the average EMI SE could still be maintained at 44.29 dB. At the same time, the composite film has good tensile properties, toughness, and ductility, whereby the tensile strength could reach 43.49 MPa, and the elongation at break reached 62.4%. The film can be expected to be an attractive candidate for practical EMI-shielding materials in flexible devices and portable wearable intelligent devices.

## Conflicts of interest

The authors declare no competing financial interest.

## Supplementary Material
